# Access to Health Care and Mental Health Among Latino Students in San Diego

**DOI:** 10.1089/heq.2019.0115

**Published:** 2020-06-16

**Authors:** Micah Gell-Redman, Lu Shi, Donglan Zhang, Ana Barbara Mungaray

**Affiliations:** ^1^ Department of Health Policy and Management, University of Georgia, Athens, Georgia, USA.; ^2^ Department of International Affairs, University of Georgia, Athens, Georgia, USA.; ^3^ College of Health, Education and Human Development, Clemson University, Clemson, South Carolina, USA.; ^4^ Facultad de Economía y Relaciones Internacionales, Universidad Autonoma de Baja California, Tijuana, Mexico.

**Keywords:** health care disparities, mental health, Latino health

## Abstract

**Purpose:** Depression during adolescence has important consequences, and Latino adolescents face different mental health challenges compared with peers from other ethnic groups. In this article we investigate whether access to a primary care physician affects the mental health of Latino high school students.

**Methods:** Our data are drawn from a unique sample survey conducted in San Diego County in 2016. Classrooms were randomly selected from six area high schools, and students currently attending school were recruited to complete the survey in class. Self-reported depression screener (Patient Health Questionnaire-2) and loneliness were collected as outcome variables, and access to a primary care physician was the main independent variable.

**Results:** Our multilevel logistic regression linking access to a physician and being at risk for major depression resulted in an odds ratio (OR) of 0.316 (95% confidence interval [CI]: 0.184–0.544), whereas the multilevel logistic regression relating access to a physician and feeling lonely resulted an OR of 0.371 (95% CI: 0.215–0.639).

**Conclusion:** This study provides evidence from a novel setting to demonstrate the link between mental health and access to health services within a minority population. Because many of the Latino adolescents in our sample come from mixed status families, this finding is particularly important in the current policy climate of the United States, in which the future of access to health care for many immigrant families is highly uncertain.

## Introduction

Public health scholars have long been aware of fundamental disparities in the physical and mental health challenges facing different populations.^1–3^ For Latino adolescents, a complex web of influences shapes those mental health challenges,^4^ including the experience of discrimination^5^ and barriers to English language proficiency.^6^ As an indicator of mental health, we focus primarily on depression. Adolescent depression is a problem with global reach, and significant consequences, including increased morbidity and risk of suicide.^7^ In the United States, scholars have long recognized that these risks may be particularly acute among some Latino youth.^8^

In this study, we consider the impact on Latino adolescents of being without easy access to a primary care physician. Existing research has focused on insurance coverage as a protective factor. Although the two concepts are distinct, here we argue that lack of insurance coverage makes accessing primary care more difficult, and thus the literature on the former topic is informative about the latter. Among other protective factors for adolescent depression,^9,10^ having insurance coverage reduces depression among low-income individuals,^11,12^ and is more generally associated with greater life satisfaction.^13^ The high rate of being uninsured among Latino youth^14^ demonstrates the barriers to health services, including access to a primary care physician, facing this population.

The key predictor variable we are interested in is access to a primary care physician. Although access to a primary care physician may be a proxy for health insurance coverage, there are also potential pathways through which it could directly impact mental health. One of these builds on an older finding in the literature, which Latino children are significantly less likely than whites to enter the pipeline of mental health care by seeking help from mental health professionals.^15^ Not having access to a primary care physician may further erode the link between adolescents and their families and health professionals. In this sense, lack of stable contact with a primary health provider could create a path dependency, leading to lower likelihood of accessing mental health services and higher incidence of depression.

To explore the role of access to health care in the mental health of Latino high school students, we gathered data from the San Diego Unified and Sweetwater school districts of San Diego County, CA. Our study focused on a context of high immigrant density and low socioeconomic status, in which the relationship between access to health services and mental health may be particularly salient. From a methodological perspective, the data gathered through our survey allow us to account for effects at the school, as well as the individual level. This research is timely, given possible changes in the policy environment that may impact the mental health of young Latinos.

## Methods

Our results are drawn from a subsample of a cross-sectional cross-border survey project that covered middle and high school students on both sides of the San Diego–Tijuana border region. Data were collected in 2016 by a binational team of student interviewers recruited from the University of California, San Diego, in the United States, and the Autonomous University of Baja California in Mexico. A multistage probability sample was used to select schools within the two districts, and classrooms were then randomly selected within schools. Approval for the study of human subjects was granted by the University of California, San Diego, and informed consent was obtained from participants in conjunction with school administrators.

Survey questionnaires were collected from all students in the sampled classrooms. Of 6465 students surveyed, we focused our analysis on students currently attending high school in San Diego County who self-identified as Latino (more precisely those who answered yes to the question, “Are you Hispanic or Latino?”). High school is a critical juncture in determining future life outcomes, including health.^16^ Our aim was to analyze the association between access to a health care provider and two self-reported mental health outcomes: depression risk and loneliness. Descriptive statistics for the sample are presented in Table 1.

**Table 1.** Table of Descriptive Statistics

Variable	Proportion	SD
Female	0.50	0.50
Born outside United States	0.13	0.33
Age	16.8	0.84
Spanish at home	0.31	0.46
Spanish and English at home	0.42	0.49
Mother does not hold diploma	0.19	0.39
Father does not hold diploma	0.21	0.40
Mother born outside United States	0.68	0.47
Father born outside United States	0.66	0.47

Lines 3 and 4 refer to the primary language that the subject speaks at home. Education variables refer to high school diplomas. All variables except age are binary indicators.

SD, standard deviation.

The key independent variable of our study is access to a primary care physician. Those who responded “no” to the questionnaire item “do you have easy access to a doctor when you need it?” were coded as 0 and those who answered “yes” were coded as 1. We note that students answering this question need not have associated this question with provision of mental health services. The question was intended simply to capture access to primary care delivered by a physician. Risk of depression was assessed using the Patient Health Questionnaire-2 (PHQ-2),^17^ a validated depression screener that has been previously administered to a similar population.^18^

Respondents answered the following two questions on a 4-point scale: “1. Over the past two weeks, how many times have you had little pleasure or interest; 2. Over the past two weeks, how many times have you felt sad, depressed, or hopeless. (1=Not at all; 2=Several days; 3=More than half the days; 4=Nearly every day).” The questions are asked without further context. The sum of the two 4-point variables was used as an integer measure of depression risk. As a PHQ-2 score of ≥3 had a sensitivity of 74% and specificity of 75% for detecting youth who met *Diagnostic and Statistical Manual of Mental Disorders* (4th edition) threshold for major depression,^19^ we coded this PHQ-2 score into a binary variable of “at risk for major depression”: those with a PHQ-2 score of 3 or greater were coded as 1 and those with a score ≤2 were coded as 0.

In addition to our main analysis of the association between physician access and risk of depression, we also show analysis in which loneliness was the dependent variable. Respondents were asked whether they agreed with the statement “I feel lonely.” Those who stated “agree” or “strongly agree” were coded as 1 for the loneliness measure and those who stated “disagree” or “strongly disagree” were coded as 0. Thus we have converted a 4-point ordinal scale to a binary measure. This measure of loneliness is not validated and is presented here as a robustness test of the relationship of primary theoretical interest.

Multilevel logistic regressions, with each school ID as the cluster variable, were used to account for the possible clustering pattern of outcome variables. The following confounding variables were used at the individual level: gender, age, paternal employment status, maternal employment status, and whether primarily speaking English at home.^20^ STATA 14's multilevel package of xtmelogit was used in the analyses.^21^ To understand the role of using multivariate multilevel models, we also run single-level simple logistic regressions of access to health care and the two mental health outcomes.

Our survey data set was not without the issue of missing data, and for variables such as parents' employment status, the proportion of missing is high: the variable of maternal employment status had 14% missing and the variable of paternal employment status had 18% missing. We decided not to perform a multiple imputation for our independent variables given the observation that imputing >10% of missing values could bias the results of statistical analyses.^22^

**FIG. 1.**
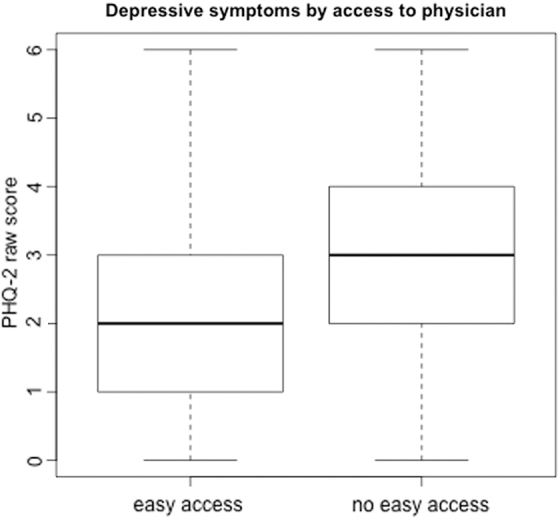
Association between easy access to primary care physician and raw PHQ-2 score. PHQ-2, Patient Health Questionnaire-2.

The specific set of individual-level confounders included here is driven primarily by the fact that few individual-level measures relevant to mental health outcomes were collected in the survey. We note here that the reported association is robust to excluding all other predictors, as well as to including only a school-level fixed effect. The bivariate relationship between easy access to a physician and risk of depression as measured by PHQ-2 is shown in Figure 1.

## Results

Our analytic sample consisted of 1067 San Diego high school students who self-identified as Latino, and who did not have missing values for any of the variables in our model. Within the analytic sample, 56 respondents answered “no” to the question about whether they had access to a doctor (5%), the mean PHQ-2 score was 2.34 and the standard deviation was 1.31, 16% of the sample reported PHQ-2 scores ≥3, the standard cutoff for being at risk of major depression. Eighteen percent reported feeling lonely.

Model results are presented in Table 2. Our single-level simple logistic regression relating access to a primary care physician and risk of major depression yielded an odds ratio (OR) of 0.481 (95% confidence interval [CI]: 0.306–0.429), whereas the single-level simple logistic regression about physician access and feeling lonely yielded an OR of 0.616 (95% CI: 0.499–0.578). Our multilevel logistic regression with depression risk as the dependent variable resulted in an OR of 0.316 (95% CI: 0.184–0.544), whereas the multilevel logistic regression with feeling lonely as the dependent variable resulted in an OR of 0.371 (95% CI: 0.215–0.639). To summarize, we find that not having easy access to a physician is associated with higher self-reported risk of depression in this sample of adolescent Latinos.

**Table 2.** Single-Level and Multilevel Logistic Regressions of Access to Health Care Services and Mental Health Outcomes

Model setup outcomes	Single-level unadjusted OR (SE)	95% CI	Adjusted OR in multilevel models^a^ (SE)	95% CI
At risk for major depression	0.481^***^ (0.045)	(0.306–0.429)	0.316^***^ (0.088)	(0.184–0.544)
Feeling lonely	0.616^***^ (0.061)	(0.499–0.578)	0.371^***^ (0.103)	(0.215–0.639)

^a^ Multilevel logistic regressions adjust for gender, age, paternal employment status, maternal employment status, and whether speaking English at home at the individual level, and account for the clustering effect at the school level.

^***^ *p*<0.001.

CI, confidence interval; OR, odds ratio; SE, standard error.

## Discussion

Our study was limited by the cross-sectional nature of our data, and by the relatively limited approach to measuring the mental health outcomes of interest (e.g., a one-item measurement of loneliness we developed for this study, and a two-item screener to identify depression risk). Our questionnaire item “do you have easy access to a doctor when you need it?” could have also missed some cases whereby the respondents did have access to nurse practitioners or physician assistants whom they did not consider as doctors. Moreover, the particular aspect of health care services measured through this question does not necessarily reflect a broader array of services and could overlap in unexpected ways with health care coverage. Although this is a potential limitation, we argue that asking students about physician access may capture access to health services more directly than a question about insurance coverage, because coverage does not necessarily guarantee access to a doctor. Finally, we note that responses to the question about easy access do not allow us to distinguish between cases of no access and cases in which access is difficult.

Despite these limitations, our study reinforces the previously documented link between mental health status and access to health care service,^11,12^ within a novel sample of Latino high school students. This extension is informative because high school students may be relatively less reliant on health care services due to their age. As such, observing the association between access to a primary care physician and mental health in this group reinforces the fundamental importance of access to health services across high-risk populations. Further studies, especially using longitudinal data and quasi-experimental design, will be needed to better understand the effect of access to health care on mental health outcomes in this population.

Our finding from this large sample in San Diego that the mean PHQ-2 score was 2.34 and 16% of the Latino high school students are at risk for major depression should be of concern for stakeholders of immigrant health and adolescent health.^19^ For context, 12% of adolescents reported a score of 3 or higher in a study of adolescents selected at random from patients of a large health system.^23^ Our sample is not representative of the U.S. Latino adolescent population, but instead of the high school students who were currently attending classes in the two school districts at the time the survey was conducted. As we already indicated, the PHQ-2 questions have been extensively validated,^24^ but to our knowledge they have not been validated in this particular population (though see, Keum et al.^25^ for study validating the PHQ-9 in a racially diverse sample of college students).

The need for future studies to supplement our results is especially pressing because of the current policy environment since 2016, in which support for health services for immigrant families is jeopardized.^26^ As Table 1 demonstrates, most of the Latino adolescents in our study come from mixed status families in which one or more parents are immigrants. Potential reductions in Medicaid coverage and ongoing changes to the Affordable Care Act all point toward increased disparities in access to care for racial and ethnic minorities. Our results further demonstrate that such policy changes could have far reaching effects not only on health care services but on health *status* as well.

## Abbreviations Used

CI: confidence interval
OR: odds ratio
PHQ-2: Patient Health Questionnaire-2
SD: standard deviation
SE: standard error
